# Intractable mechanical hemolytic anemia complicating mitral valve surgery: a case series study

**DOI:** 10.1186/s12872-020-01382-8

**Published:** 2020-03-03

**Authors:** Jin Wang, Hanlin Zhang, Hongyang Fan, Kang Chen, Yuelun Zhang, Kaicheng Song, Hushan Ao, Chunhua Yu

**Affiliations:** 1 Department of Anesthesiology, Peking Union Medical College Hospital, Chinese Academy of Medical Sciences, 100730, No.1 Shuaifu Yuan, Dongcheng District, Beijing, China; 2 Peking Union Medical College, Chinese Academy of Medical Sciences, Beijing, China; 3 Department of Surgery, Fu Wai Hospital, Chinese Academy of Medical Sciences, Beijing, China; 4 Department of Anesthesiology, Fu Wai Hospital, Chinese Academy of Medical Sciences, 100037, No.167 North Lishi Road, Xicheng District, Beijing, China

**Keywords:** Mechanical hemolysis, Mitral valve surgery, Perioperative management

## Abstract

**Background:**

Intractable, mechanical hemolytic anemia (IMHA) is a rare catastrophic complication following mitral valve surgery. We analyzed patient characteristics and IMHA management by reoperations after mitral valve surgery.

**Methods:**

We collected medical records from mitral valve patients requiring reoperation due to IMHA. Inclusion criteria: hemoglobin < 100 g/L; positive hemolysis tests and echocardiography results; and exclusion of other hemolysis causes.

**Results:**

Data from 25 IMHA cases included 10 (40%) early onset (1.3 (0.3,3.0) months) and 15 (60%) late onset (120 (24,204) months) cases. Early IMHA etiologies included surgical defects (6, 60%), uncontrolled infection (3, 30%) and Bechet’s disease (1, 10%). Late IMHA etiologies included degeneration (13, 87%), new infection (1, 7%) and trauma (1, 7%). There were more mechanical valves (15, 88%) than bio-valves (2, 12%); the main valvular dysfunction was paravalvular leak (16, 64%). IMHA manifestations included jaundice (18, 72%), dark urine (21, 84%), heart failure (16, 64%), acute kidney injury (11, 44%), hepatomegaly (15, 60%), splenomegaly (15, 60%) and pancreatitis (1, 4%). Laboratory results showed decreased hemoglobin (70 ± 14 g/L) and increased bilirubin (72 ± 57 μmol/L), lactate dehydrogenase (2607 ± 2142 IU/L) and creatinine (136 ± 101 μmol/L) levels. Creatinine level negatively correlated with hemoglobin level (B = -3.33, S.E. B = 1.31, Exp(B) = 368.15, *P* = 0.018). Preoperative medications included iron supplements (20, 80%), erythropoietin (16, 64%) and beta-blocker (22, 88%). Two patients died of cardiac causes before reoperation. The other 23 underwent reoperation with long surgical times (aortic cross clamp 124 ± 50 min, cardiopulmonary bypass 182 ± 69 min) and blood transfusions (red blood cells 6 (6, 8) units, plasma 600 (400,800) ml, platelet 1(0,2) units). Postoperative complications included cardiac dysfunction (5, 22%), arrhythmia (10, 43%), sepsis (6, 26%), pulmonary infection (5, 22%), gastrointestinal bleeding (3, 13%), cerebral hemorrhage (2, 9%), chronic renal dysfunction (1, 4%) and surgical hemorrhage (1, 4%). Five (33%) patients died after reoperation from cardiac dysfunction (3, 60%), septic shock (1, 20%) and self-discharge (1, 20%).

**Conclusions:**

IMHA induces severe multi-organ dysfunction, contributing to high mortality. Perioperative management should focus on etiological treatment, organ protection, and blood management.

## Background

Mechanical hemolysis is caused by prosthetic valve dysfunction. The pathogenesis is hydrodynamic shearing of the erythrocytes by turbulent flow [1].

Mild mechanical hemolytic anemia can be treated with beta-blockers, iron supplements, and pentoxifylline [2]. The intractable type requires repeated blood transfusions often necessitating reoperation [1].

Intractable mechanical hemolytic anemia (IMHA) following mitral valve surgery has a reported incidence of less than 1% and a reoperation rate of 74% [2–5]. Though procedural risk factors were reported in both surgery and interventional cardiology [4, 6–9], patient characteristics and perioperative management during reoperation are rarely discussed.

This study reviewed a series of IMHA reoperation cases from two closely collaborated hospitals. Patients’ clinical characteristics and perioperative management were discussed.

## Methods

### Patient selection

We reviewed medical records of patients admitted for mitral valve surgery from January 2008 to December 2018 in two collaborating hospitals. Data from patients requiring reoperations due to IMHA were collected. IMHA patients were selected based on the following criteria: (1) moderate-to-severe anemia (hemoglobin, Hb < 100 g/L) with positive hemolysis test results; (2) mitral valve dysfunction with echocardiography; and (3) exclusion of other possible hemolytic anemia causes.

### Perioperative management

Hemolytic anemia patients were admitted to the departments of hematology, infectious disease, cardiology and cardiovascular surgery.

Hematological and etiological tests were performed either in the hospitals’ laboratory or the affiliated national academic institution. We used a blood smear test to detect erythrocyte rupture and deformation. Hemoglobin, reticulocyte, free hemoglobin, haptoglobin, total (TBil) and direct (DBil) bilirubin, lactate dehydrogenase (LDH), urine hemoglobin, urine hemosiderin and urobilinogen were tested for the type, duration, and severity of hemolysis. Etiological tests included the Coombs test, Ham’s test, and glucose-6-phosphodehydrogenase and pyruvate kinase activity tests, in addition to pathogenic tests and echocardiography.

Reoperation was arranged if IMHA was considered. Preoperative medication included iron supplements, erythropoietin, and beta-blockers. Warfarin was replaced by low molecular weight heparin in mechanical valve patients. Red blood cells (RBC) and plasma transfusion were considered if patients had very severe anemia. Chronic patients receiving repeated transfusions were monitored for hemochromatosis.

Major preoperative organ dysfunction included heart failure, acute kidney injury, hepatomegaly and splenomegaly. Hepatomegaly and splenomegaly were examined using ultrasonography by certified clinicians. Echocardiography was routinely performed as follows: (1) after hospital admission but before reoperation, (2) before and after bypass during reoperation, (3) after reoperation but before hospital discharge. Bedside echocardiography could be performed when emergent cardiac events were suspected.

Reoperations were performed under general anesthesia and cardiopulmonary bypass (CPB). Intraoperative monitoring included electrocardiogram, oxygenation, invasive arterial blood pressure, central venous pressure and core temperature. Hepatic and renal protective anesthetics were used, and heparin was used as the bypass anticoagulants. Vasoactive agents were adjusted based on patients’ vital signs. Patients with infection were administered pathogen-sensitive antibiotics and usually required large doses of peripheral vasoconstrictor. Patients with autoimmune disease were administered individualized intravenous steroids doses. Perioperative adrenal insufficiency and vasoplegia were considered if intractable hypotension occurred.

Patients were sent to the intensive care unit (ICU) after reoperation. Routine intensive care included mechanical ventilation, sedatives, vasoactive agents, anti-arrhythmic agents, furosemide, antibiotics, steroids, hepatic and gastric protective agents. Continuous cardiac output monitoring was established to guide vasoactive agent dosing, and intra-aortic balloon pump (IABP) use was considered if severe cardiac dysfunction occurred. Continuous renal replacement therapy (CRRT) was administered if severe renal dysfunction or hyperkalemia occurred.

Iron supplements and erythropoietin were continued after reoperation until self-compensation. Blood products were transfused based on patients’ hemoglobin, platelet, and coagulation test results. Heparin was used as an early postoperative anticoagulant under the guidance of activated clotting time and replaced by warfarin later before hospital discharge when using a mechanical valve. The patients’ feces and pupils were intermittently checked to identify gastrointestinal and intracranial complications, respectively.

Patients were sent back to the ward after weaning of ventilator and vasoactive agents. Hospital discharge was considered after bedside activities.

### Data collection

Patient demographics, clinical characteristics and perioperative data were collected as follows: (1) demographic data: age, gender, body mass index (BMI), smoking and drinking history, history of hypertension, diabetes mellitus, stroke, atrial fibrillation and coronary artery disease; (2) first and reoperation data: primary heart valve disease, reoperation etiology and valve dysfunction type, first and reoperation type, first and reoperation valve type and brand, onset time of hemolysis, (3) perioperative data during reoperation: symptoms and signs including jaundice, dark urine, heart failure, acute kidney injury, hepatomegaly and splenomegaly; laboratory results including Hb, TBil, DBil, LDH and creatinine (Cr, μmol/L) level; mitral valve echocardiography manifestations; intraoperative data including aortic cross-clamp (ACC) time, CPB time, allogeneic blood transfusion volume, urine output and vasoactive agents use; postoperative complications including cardiac, cerebral, renal and gastrointestinal dysfunction, mechanical circulatory support device use and outcomes including ventilator hours, length of ICU and hospital stay, perioperative mortality and causes of death.

### Statistical analysis

Statistical analysis was performed using SPSS for Mac version 23.0 (IBM Corp., Armonk, NY, USA). Normality was calculated using the Shapiro–Wilk test. Normally distributed variables are expressed as mean ± SD, non-normally distributed variables are expressed as median (quartile) and categorical variables are expressed as frequency (percentage). Correlations were analyzed using Pearson’s test and linear regression. All tests were two-tailed and *P*-values less than 0.05 were considered statistically significant.

## Results

### Participants

A total of 2778 mitral valve patients’ medical records were searched. Of these 1241 had a complete follow-up record; 12 of these developed IMHA and were admitted for reoperation. Another five patients were suspected of having IMHA; however, their reoperation data were unobtainable because they were not readmitted to the studied hospitals. Of the 1537 patients without complete follow-up records, we identified another 13 IMHA patients admitted for reoperations in the studied hospitals. Their first operations were performed previously or in other hospitals; therefore, no follow-up work was performed. Finally, reoperation data for 25 patients were collected.

Among the 25 patients, ten (40%) developed IMHA early either in the hospital or after discharge but before returning to daily life. The other 15 (60%) developed IMHA years later after returning to daily life. We listed data of the early and late IMHA patients separately, because they have different characteristics (Tables 1, 2, 3, 4, 5 and 6).

**Table 1:** Baseline characteristics of IMHA patients

	Early IMHA	Late IMHA	Total
*Demographics*			
Age (y/o)	49 ± 14	55 ± 10	53 ± 12
Male (n,%)	7 (70)	12 (80)	19 (76)
BMI (kg•m^2^)	22.60 ± 3.09	22.50 ± 3.21	22.54 ± 3.16
Smoking (n,%)	1 (10)	9 (60)	10 (40)
Drinking (n,%)	1 (10)	5 (33)	6 (24)
*Comorbidities*			
Hypertension (n,%)	3 (30)	3 (20)	6 (24)
Diabetes (n,%)	2 (20)	0 (0)	2 (8)
Stroke (n,%)	2 (20)	1 (7)	3 (12)
Atrial fibrillation (n,%)	0 (0)	6 (40)	6 (24)
CAD (n,%)	2 (20)	1 (7)	3 (12)

**Table 2:** First operation data of IMHA patients

	Early IMHA	Late IMHA	Total
*Primary mitral valve disease*			
Rheumatic	2 (20)	11 (73)	13 (52)
Infection	4 (40)	1 (7)	5 (20)
Others	4 (40)	3 (20)	7 (28)
*First operation*			
Mitral valve repair	6 (60)	2 (13)	8 (32)
Mitral valve replacement	4 (40)	13 (87)	17 (68)
*Prosthetic valve type*			
Bio-valve	1 (25)	1 (8)	2 (12)
Mechanical valve	3 (75)	12 (92)	15 (88)
Valve brand			
CarboMedics	2 (50)	4 (31)	6 (35)
Medtronics	1 (25)	2 (15)	3 (18)
St Jude	0 (0)	4 (31)	4 (24)
Sorin	0 (0)	1 (8)	1 (6)
Unknown	1 (25)	2 (15)	3 (18)

**Table 3:** Reoperation data of IMHA patients

	Early IMHA	Late IMHA	Total
*Onset time after the first mitral valve operation*			
0–6 months	10 (100)	0 (0)	10 (40)
6–12 months	0 (0)	0 (0)	0 (0)
1–10 years	0 (0)	7 (47)	7 (28)
10–20 years	0 (0)	6 (40)	6 (24)
20–30 years	0 (0)	2 (13)	2 (8)
*Reoperation etiologies*			
Surgical defects	6 (60)	0 (0)	6 (24)
Degeneration	0 (0)	13 (87)	13 (52)
Infection	3 (30)	1 (7)	4 (16)
Vasculitis	1 (10)	0 (0)	1 (4)
Trauma	0 (0)	1 (7)	1 (4)
*Mitral valve dysfunction type*			
Paravalvular leak	4 (40)	12 (80)	16 (64)
Open-closure dysfunction	4 (40)	3 (20)	7 (28)
Perforation	2 (20)	0 (0)	2 (8)
Paravalvular leakage location (midpoint)			
2–3 o’clock	2 (50)	4 (33)	6 (38)
5–8 o’clock	2 (50)	3 (25)	5 (31)
10–12 o’clock	0 (0)	3 (25)	3 (19)
Unknown	0 (0)	2 (17)	2 (13)
*Reoperation*			
Mitral valve replacement	8 (80)	14 (93)	22 (88)
Mitral valve repair	1 (10)	0 (0)	1 (4)
Died before reoperation	1 (10)	1 (7)	2 (8)
Valve brand			
CarboMedics	2 (25)	6 (43)	8 (36)
Medtronics	2 (25)	3 (21)	5 (23)
St Jude	2 (25)	2 (14)	4 (18)
Sorin	0 (0)	1 (7)	1 (5)
Unknown	2 (25)	2 (14)	4 (18)

**Table 4:** Pre-reoperation data of IMHA patients

	Early IMHA	Late IMHA	Total
*Symptoms and signs*			
Jaundice	6 (60)	12 (80)	18 (72)
Dark Urine	8 (80)	13 (87)	21 (84)
Heart failure	3 (30)	13 (87)	16 (64)
Acute kidney injury	4 (40)	7 (47)	11 (44)
Hepatomegaly	3 (30)	12 (80)	15 (60)
Splenomegaly	3 (30)	12 (80)	15 (60)
*Laboratory results*			
Hb (g/L)	68 ± 14	72 ± 16	70 ± 14
Cr (μmol/L)	127 ± 95	142 ± 121	136 ± 101
Tbil (μmol/L)	41 ± 19	89 ± 62	72 ± 57
Dbil (μmol/L)	13 ± 7	18 ± 8	16 ± 7
LDH (IU/L)	1623 ± 1009	3263 ± 2462	2607 ± 2142
*Mitral valve echo parameters*			
Flow rate (m/s)	2.3 ± 0.6	2.6 ± 0.4	2.5 ± 0.5
Peak pressure (mmHg)	23.3 ± 11.4	27.2 ± 9.3	25.6 ± 10.3
Regurgitation (1+/2+/3+)	1/3/6	1/10/4	2/13/10
Pre-op transfusion volume			
RBC (U)	1 (0,2)	5 (2,8)	2 (1,7)
Plasma (ml)	0 (0, 100)	100 (0,400)	0 (0,400)
Pre-op medication			
Beta-blocker	9 (90)	13 (87)	22 (88)
Iron supplements	8 (80)	12 (80)	20 (80)
Erythropoietin	6 (60)	10 (67)	16 (64)

**Table 5:** Intra-reoperation data of IMHA patients

	Early IMHA	Late IMHA	Total
ACC time (min)	121 ± 61	125 ± 45	124 ± 50
CPB time (min)	186 ± 69	180 ± 69	182 ± 69
RBC (U)	8 (5, 8)	6 (6,10)	6 (6,8)
Plasma (ml)	800 (400, 800)	600 (400, 700)	600 (400, 800)
Platelet (U)	1 (0,2)	1 (1,2)	1 (0,2)
Urine (ml)	686 ± 448	394 ± 148	472 ± 326
Epinephrine	5 (56)	7 (50)	12 (52)
Norepinephrine	5 (56)	7 (50)	12 (52)
Dopamine	4 (44)	7 (50)	11 (48)
Dobutamine	1 (11)	2 (14)	3 (13)
Amiodarone	5 (56)	10 (71)	15 (65)

**Table 6:** Complications and outcomes of IMHA patients after reoperation

	Early IMHA	Late IMHA	Total
*Complications*			
Low cardiac output	1 (11)	4 (29)	5 (22)
Arrhythmia	2 (22)	8 (57)	10 (43)
Septic shock	1 (11)	5 (36)	6 (26)
Pulmonary infection	2 (22)	3 (21)	5 (22)
Gastrointestinal bleeding	0 (0)	3 (21)	3 (13)
Cerebral hemorrhage	1 (11)	1 (7)	2 (9)
Chronic renal dysfunction	0 (0)	1 (7)	1 (4)
Surgical hemorrhage	0 (0)	1 (7)	1 (4)
Tracheotomy	0 (0)	2 (14)	2 (9)
CRRT	3 (33)	7 (50)	10 (43)
IABP	0 (0)	2 (14)	2 (9)
ECMO	0 (0)	0 (0)	0 (0)
*Outcomes*			
Ventilator (hours)	35 ± 30	413 ± 376	259 ± 342
Length of ICU stay (d)	4 ± 2	9 ± 6	7 ± 5
Length of hospital stay (d)	30 ± 20	37 ± 16	31 ± 17
Mortality (n,%)	2 (20)	5 (33)	7 (28)

### Demographics

Demographic data are displayed in Table 1. The majority of the IMHA patients were middle aged (53 ± 12 years old) and male (19,76%). The late IMHA group included many smokers (early: 1,10% versus late: 9,60%). Major comorbidities included hypertension (6, 24%), atrial fibrillation (6, 24%), coronary artery disease (3, 12%), stroke (3, 12%) and diabetes mellitus (2, 8%). All atrial fibrillation patients were in the late group (early 0, 0% versus late 6, 40%).

### First operation

The first operation data are displayed in Table 2. Early IMHA patients had various primary heart diseases, including two (20%) rheumatic valvular diseases, four (40%) infections, one (10%) vasculitis and three (30%) congenital lesions. The majority of the late IMHA patients had rheumatic heart diseases (11, 73%). Early IMHA patients received more mitral valve repair (6, 60%) than replacement (4, 40%) surgeries. The majority of the late IMHA patients received mitral valve replacement (13, 87%). For mitral valve replacement surgeries, the prosthetic valves were mainly mechanical valves (15, 88%) in both early (3, 75%) and (12, 92%) late groups. Various valvular brands were used, including CarboMedics (6, 35%), Medtronics (3, 18%), St Jude (4, 24%), Sorin (1, 6%), and unknown (3, 18%).

### Reoperation

Reoperation data are displayed in Table 3. IMHA developed postoperatively from as early as 11 days to as late as 30 years. Early IMHA developed from postoperative 11 days to 6 months, with a median (quartile) of 1.3 (0.3, 3.0) months. Late IMHA developed from postoperative 1 to 30 years, with a median (quartile) of 120 (24, 204) months.

Early IMHA was mainly attributed to surgical defects (6, 60%), including suture dehiscence (4, 67%), valve prolapse (1, 17%) and leaflet retraction (1, 17%). Other early IMHA causes included uncontrolled infection (3, 30%) and Behcet’s disease (1, 10%). Thirteen (87%) late group patients developed IMHA gradually without apparent trigger events, and their IMHA was considered due to degeneration of either the valves or the tissues. The other late IMHA causes included new infections (1, 7%) and trauma (1, 7%).

The majority (16, 64%) of mitral valve dysfunction were paravalvular leaks (early 4, 40% versus late 12, 80%), the recorded leakage was at 2–3, 5–8 and 10–12 o’clock locations. Other dysfunctions included open-closure dysfunction (early 4, 40% versus 3, 20%) and perforation (early 2, 20% versus late 0, 0%). Reoperations were mainly mitral valve replacement (early 8, 80% versus late 14, 93%). Twenty-three patients underwent reoperation and two died before reoperation.

### Preoperative data

The main manifestations of IMHA included jaundice (18,72%), dark urine (21, 84%), heart failure (16, 64%), acute kidney injury (11, 44%), hepatomegaly (15, 60%) and splenomegaly (15, 60%). One patient developed pancreatitis, which was relatively rare. Many patients of the late IMHA group had heart failure (early 3, 30% versus 13, 87%).

Important blood test results included decreased Hb (70 ± 14 g/L) levels, increased TBil (72 ± 57 μmol/L), DBil (16 ± 7 μmol/L), LDH (2607 ± 2142 IU/L) and Cr (136 ± 101 μmol/L) levels. Linear regression revealed a negative correlation between Hb and Cr level (B = -3.33, S.E. B = 1.31, Exp (B) = 368.15, *P* = 0.018), meaning that for every 1 g/L Hb decrease, Cr increases by 3.33 μmol/L. Many late IMHA patients had critical LDH level (early: 1623 ± 1009 IU/L versus late: 3263 ± 2462 IU/L), which correlated with hemolysis severity.

Echocardiography showed that paravalvular leaks and perforations were 2 mm to 15 mm long. The malfunctioned valve created high speed, eccentric regurgitation jet, which sheared the erythrocytes. Open-closure dysfunction created fragmented, accelerating flow jet colliding with the valve or the ceiling of the left atrial wall, which tore the erythrocytes. All types of dysfunction had significantly increased blood flow rate (range: 1.5–3.2 m/s) and peak pressure (range: 9–42 mmHg). However, regurgitations did not need to be severe, both groups had one patient with mild regurgitation.

Iron supplements (20, 80%), erythropoietin (16, 64%), and beta-blocker (22, 88%) were administered to the majority of patients. A pancreatitis patient also received fasting, anti-acids and somatostatin treatment. Some late IMHA patients received 8–10 units of RBC transfused preoperatively, which is relatively large. One of them developed secondary hemosiderin due to repeated transfusion and reoperation was arranged sooner.

### Intraoperative data

The ACC and CPB time during reoperations were 124 ± 50 min and 182 ± 69 min, respectively. Blood transfusions included 6 (6, 8) units RBC, 600 (400, 800) ml plasma and 1 (0, 2) units of platelet. Many late IMHA patients had poor urine output (early 686 ± 448 versus late 394 ± 148 ml). Vasoactive agents used included epinephrine (12, 52%), norepinephrine (12, 52%), dopamine (11, 48%), dobutamine (3, 13%) and amiodarone (15, 65%). Pituitrin was administered to two patients that were suspected of vasoplegia.

### Postoperative data

Major postoperative complications included cardiac dysfunction (5, 22%), arrhythmia (10, 43%), septic shock (6, 26%), pulmonary infection (5, 22%), gastrointestinal bleeding (3, 13%), cerebral hemorrhage (2, 9%), chronic renal dysfunction (1, 4%) and surgical hemorrhage (1, 4%). Ten patients (43%) underwent CRRT because of severe renal dysfunction and two patients (9%) underwent IABP because of severe cardiac dysfunction.

The mean postoperative ventilator time was 259 ± 342 h, mean length of ICU and hospital stays were 7 ± 5 and 31 ± 17 days, respectively. Two patients died before reoperation: one died in the hospital due to malignant arrhythmia, the other self-discharged due to financial issues and died of heart failure after 22 days.

Of the remaining 23 patients, five (22%) died after reoperation within 3 to 42 days. Major causes of death were cardiac dysfunction (3, 60%) and infection-induced multiple organ dysfunction syndrome (1, 20%). One (20%) patient self-discharged after reoperation due to financial tissues and died of unknown causes.

## Discussion

### Key results

This study describes the clinical characteristics and perioperative management of IMHA patients during reoperation. IMHA occurred days or years after the first operation. Early and late IMHA had different etiologies and surgery types, but similar prosthetic valve types and dysfunction patterns. IMHA could be both intra- and extravascular hemolysis, with either normocytic, normochromic or microcytic, hypo-chromatic anemia. Major involved systems included hematology, coagulation, cardiovascular, kidney, liver, spleen, and pancreas. Perioperative management should focus on etiological treatment, organ protection, and blood management.

### Interpretation

Early and late IMHA had different etiologies and surgery types, but similar prosthetic valve types and malfunction patterns. Previous studies reported many early IMHA cases. A nine-patient retrospective study reported that IMHA developed from 1 to 44 days postoperatively [10]. Two studies on mitral valve surgeries reported that IMHA developed within an average of postoperative 3 months [7, 11]. In our study population, all early IMHA developed within 6-months post-operation, which should be a close follow-up time frame.

The differences in primary heart disease and the first operation type explained the differences in IMHA etiology. The majority (80%) of the early IMHA patients had non-rheumatic heart diseases, including infection, vasculitis and congenital defects; 60% of them received mitral valve repair during the first operation. Therefore, early IMHA occurred due to surgical defects (60%) from an unsuccessful repair, or perioperative care problems (40%), such as an uncontrolled infection or autoimmune disease.

The majority (73%) of the late IMHA patients had rheumatic heart diseases and had undergone mitral valve replacement surgeries (87%) previously; therefore their IMHAs were primarily (87%) caused by tissue degeneration or valve failure over time.

Previous studies reported a series of improper surgical techniques including continuous suture technique, papillary muscle approximation technique and unmatched prosthetic valve size [4, 6–8]. With improvements in surgical technique and prosthetic valve advancement, early IMHA cases are fewer than late IMHA cases [12, 13].

A mechanical valve has a higher IMHA risk than bio-valves; the main malfunction pattern was paravalvular leak [2, 3, 12, 14]. A previous study found that mitral valve replacement and mechanical prosthesis were independent risk factors of subclinical hemolysis [14]. Our study also found that both early and late IMHA patients receiving mitral valve replacement during the first operation had high mechanical valve rate (75% versus 92%) and paravalvular leak accounts for 64% of the IMHA. Furthermore, IMHA patients generally had very severe valvular damages; therefore, irrespective of the first operations, the majority of reoperations were mitral valve replacement in both the early (80%) and late (93%) groups.

IMHA can be intravascular or mixed hemolysis. In our studied population, five patients had increased free hemoglobin and decreased haptoglobin, but normal serum bilirubin levels, considered to indicate pure intravascular hemolysis. The other patients also had extravascular hemolysis manifestations such as increased bilirubin levels, hepatomegaly, and splenomegaly. The potential explanation was that prosthetic valve dysfunction generates high speed and pressure gradient blood flow with high shear stress [15]. When this stress directly ruptured the erythrocytes, intravascular hemolysis occurs. When this stress only deformed the erythrocytes or compromised their membranes, it would be recognized and digested by macrophages in the liver and spleen, causing extravascular hemolysis. Both intravascular and extravascular hemolysis can be aggravated by cardiac-burden factors such as increased physical activity or tachycardia, which should be promptly treated or prevented.

IMHA can be either normocytic, normochromic, or microcytic, hypochromic anemia, because long-term hemolysis induces erythrocytosis and urine loses iron. Iron supplements are always required. In our study population, 80% of the patients had microcytic hypochromic anemia and prescribed with iron supplements. Notably, repeated transfusion may induce hemochromatosis. One of our patients developed secondary hemochromatosis due to repeated transfusion. Fortunately, he only had increased transferrin and ferritin level without apparent organ dysfunction. However, long-term patients may present with cardiac, hepatic, and endocrine dysfunctions. Severe cases may develop diastolic dysfunction and arrhythmias, threatening patient life [16]. As for hemochromatosis treatment, phlebotomy and chelate treatment were commonly used to reduce iron load; however, phlebotomy should not be performed in IMHA patients because it can aggravate anemia.

Another common organ dysfunction of IMHA is acute kidney injury. Forty-four percent of our study patients developed acute kidney injury and one high-risk patient with prior nephritis history developed chronic renal dysfunction despite continuous postoperative hemofiltration. The underlying mechanisms are multiple, including the direct cytotoxicity of heme to tubular cells, hemosiderin pigment accumulation in proximal tubular epithelial cells, and obstructive casts with Hb precipitation [17, 18]. Hemoglobinuria and hemosiderinuria are often considered to be well-tolerated benign conditions. However, patients with previous tubulointerstitial disease, long-standing hypertension, and atherosclerotic vascular disease have increased susceptibility to the injurious effects of heme proteins and developing chronic renal dysfunction [17, 19].

One patient developed pancreatitis preoperatively, a relatively rare complication of hemolysis. Similar cases have been reported with ventricular assist device use, transfusion and hemolytic, elevated liver enzyme, and low platelet syndrome [20]. Heme also plays an important role in the disease process [21]. The underlying mechanisms included neutrophil activation, chemo-attraction, oxidative, microcirculatory disturbance, increased inflammatory and immunoregulatory cytokines.

According to previous data, the mortality of redo mitral valve surgery was around 12% [22]. In our case series, seven patients died perioperatively, a relatively high proportion. Perioperative management should focus on etiological treatment, organ protection, and blood management. Several patients developed IMHA due to uncontrolled infection or autoimmune disease, which might be avoided if appropriate antibiotics or steroids had been given with the right doses at the right time. Major organ protection should focus on the heart, kidney, liver, and the hematological and coagulation systems, since major postoperative complications and circulatory supporting devices applications are related to these organ dysfunctions. Some late IMHA patients were critical and required postoperative cardiac and renal supporting devices. These patients had long ventilator hours and ICU and hospital stay.

Another important perioperative management issue is blood management [1]. Both early and late group patients had a preoperative blood transfusion to improve severe anemia (Hb < 50 g/L). However, preoperative transfusion should not be too aggressive, because rather than transiently improving anemia, the transfused erythrocytes would be quickly sheared and ruptured by the malfunctioning valve, further aggravating hemolysis and related organ dysfunctions. Other blood products such as plasma and platelet should also be considered to correct coagulopathy, because many patients had hepatic and splenic dysfunction, and because of the use of anticoagulants, such as warfarin and heparin.

### Limitation

The major limitation of our study is the small case number due to the low incidence of IMHA; therefore statistical analysis was not fully conducted. More cases are needed to present this special clinical situation realistically.

## Conclusions

IMHA is a rare but catastrophic complication following mitral valve surgery. Reoperation for IMHA patients remains a challenge for perioperative care teams in both the surgical and interventional cardiac areas. Clinicians should be familiar with the disease process and patient characteristics. Perioperative care should focus on etiological treatment, major organ protection and blood management. IMHA patients are at high risk of perioperative death; therefore, clinicians should be aware of the risks and be fully prepared for reoperations.

## Data Availability

The datasets used and analyzed during the current study are available from the corresponding author on reasonable request.

## Abbreviations

ACC: Aortic cross clamp
BMI: Body mass index
CAD: Coronary artery disease
CPB: Cardiopulmonary bypass
Cr: Creatinine
CRRT: Continuous renal replacement therapy
ECMO: Extracorporeal membrane oxygenation
Hb: Hemoglobin
IABP: Intra-aortic balloon pump
ICU: Intensive care unit
IMHA: Intractable mechanical hemolytic anemia
LDH: Lactate dehydrogenase
RBC: Red blood cell
